# Stem Cell Factor SOX9 Interacts with a Cell Death Regulator RIPK1 and Results in Escape of Cancer Stem Cell Death

**DOI:** 10.3390/cells11030363

**Published:** 2022-01-21

**Authors:** Mijung Oh, Chaeyeon Son, Seung Bae Rho, Minjeong Kim, Kyoungsook Park, Sang Yong Song

**Affiliations:** 1 Medical Research Center, Sungkyunkwan University School of Medicine, Suwon 16419, Korea; mijung.oh1@gmail.com (M.O.); snicki0203@gmail.com (C.S.); 2 Department of Pathology and Translational Genomics, Samsung Medical Center, Sungkyunkwan University School of Medicine, Seoul 06351, Korea; claramj.kim@sbri.co.kr; 3 Division of Translational Science, Research Institute, National Cancer Center, Goyang 10408, Korea; sbrho@ncc.re.kr

**Keywords:** SOX9, RIPK1, cancer stemness, cancer cell death, protein-protein interaction, HGOC

## Abstract

High-grade ovarian cancer (HGOC) is the most lethal gynecological cancer, with high metastasis and recurrence. Cancer stem cells (CSCs) are responsible for its apoptosis resistance, cancer metastasis, and recurrence. Thus, targeting CSCs would be a promising strategy for overcoming chemotherapy resistance and improving patient prognosis in HGOC. Among upregulated oncogenic proteins in HGOC, we found that transcription factor SOX9 showed a strong correlation with stemness-regulating ALDH1A1 and was localized predominantly in the cytoplasm of HGOC with lymph node metastasis. In order to address the role of unusual cytoplasmic SOX9 and to explore its underlying mechanism in HGOC malignancy, a Y2H assay was used to identify a necroptotic cell death-associated cytoplasmic protein, receptor-interacting serine/threonine protein kinase 1 (RIPK1), as a novel SOX9-interacting partner and further mapped their respective interacting domains. The C-terminal region containing the transactivation domain of SOX9 interacted with the death domain of R1PK1. Consistent with its stemness-promoting function, SOX9 knockdown in vitro resulted in changes in cell morphology, cell cycle, stem cell marker expression, cell invasion, and sphere formation. Furthermore, in vivo knockdown completely inhibited tumor growth in mouse xenograft model. We propose that cytoplasmic SOX9-mediated cell death suppression would contribute to cancer stem cell survival in HGOC.

## 1. Introduction

High-grade ovarian carcinoma (HGOC) represents the most lethal gynecological malignancy and has the worst prognosis due to its high apoptosis resistance and metastasis [1,2]. Apoptosis resistance develops in HGOC and increases the risk of cancer recurrence, resulting in a high mortality rate [3]. Metastasis and recurrence of HGOC are associated with the spread of cancer stem-like cells (CSCs) from the primary tumor to the peritoneal cavity [4]. These CSCs are exclusively tumorigenic and major drivers of tumor formation, progression, metastasis, and apoptosis resistance to chemotherapy and radiation, eventually resulting in cancer recurrence [5] by escaping programmed cell death in various types of cancers [6,7,8]. Accordingly, platinum-based anticancer drugs that have been used as a first treatment of HGOC patients failed to eliminate apoptosis-resistant CSCs and resulted in tumor metastasis and recurrence, finally resulting in poor clinical outcomes [2,9]. Therefore, the development of a promising therapeutic strategy for treatment of HGOC is in high demand and should be directed to target CSCs.

Since apoptosis resistance is a major obstacle resulting in chemotherapy failure during ovarian cancer treatment, bypassing the apoptotic pathway would be a promising strategy to induce CSCs death in HGOC. In order to overcome CSC-mediated apoptosis resistance, necroptosis has emerged as a promising alternative mode of cancer cell death in ovarian cancer [8,10]. Necroptosis is a necrotic cell death modality regulated in a caspase-independent fashion and mediated by receptor-interacting protein kinase 1 (RIPK1), receptor-interacting protein kinase 3 (RIPK3), and mixed lineage kinase domain-like MLKL pseudokinase [7,11]. Necroptosis has been suggested to play a pivotal role in the regulation of cancer biology. Inducing and/or manipulating necroptosis in anti-cancer therapies represent a promising therapeutic approach for bypassing apoptosis resistance, serving as an alternative method for eliminating apoptosis-resistant cancer cells [12].

SOX9 is a member of the SOX family, sex-determining region Y (SRY)-related high mobility group (HMG) box (SOX) transcription factors, and is critical for embryogenesis and tissue homogenesis [13]. Accumulating experimental and clinical data reveal an important role of SOX9 in tumor development due to its overexpression and strong correlation with tumor progression and metastasis in a wide range of human cancers including ovarian cancer [14,15]. In addition, SOX9 was reported as an indicator of poor chemotherapy response and low survival rate in HGOC [16] and pancreatic cancer cells [17]. These findings have provided strong evidence of the role of transcription factor SOX9 as a regulator of proliferation and self-renewal in normal development and cancer progression. However, its role in the regulation of direct cancer cell death remains unknown.

Here, we aimed to explore the role and underlying mechanism of a SOX9 in the regulation of cancer cell death using in vitro and in vivo approaches. We discovered elevated predominant cytoplasmic SOX9 expression in HGOC and found a strong correlation between cytoplasmic SOX9 and ALDH1A1 expression only in HGOC with lymph node metastasis. In order to explore a novel function of cytoplasmic SOX9 protein, we set out to identify cytoplasmic SOX9-interacting proteins and identified programmed cell death-regulating, receptor-interacting, and serine-threonine protein kinase 1 (RIPK1) as a novel SOX9-interacting partner. Our findings suggest an unidentified function of cytoplasmic SOX9 protein in the suppression of cancer stem cell death in HGOC. We propose an underlying mechanism of cytoplasmic SOX9-mediated suppression of cancer stem cell death, subsequently resulting in ovarian cancer survival in HGOC.

## 2. Materials and Methods

### 2.1. Cell Lines, Reagents, and Chemicals

All cell culture materials were purchased from GIBCO (Carlsbad, CA, USA). EZ-Cytox Cell Viability Assay Kit was purchased from Daeil Lab Service (EZ-3000, Seoul, Korea). Puromycin was purchased from Thermo Fisher Scientific (A11138, Waltham, MA, USA). Annexin-V staining kit was purchased from BD Biosciences (556547, Franklin Lakes, NJ, USA). RNase A (R6135) and propidium iodide (PI) solution (P4864), used for the cell cycle assay, were purchased from Sigma-Aldrich (St. Louis, MO, USA). Sox9 (H-90) antibody was obtained from Santa Cruz Biotechnology (sc-20095, Dallas, TX, USA). DMSO (D2650), and cisplatin (1134357) were purchased from Sigma-Aldrich. Cisplatin was freshly prepared as a 100-millimolar solution in DMSO and used immediately for each experiment. The ALDEFLUOR kit was obtained from STEMCELL Technologies Inc. (01700, Vancouver, BC, Canada). The human ovarian cancer cell line (MDAH-2774) was purchased from American Type Culture Collection (ATCC) (Manassas, VA, USA) and cultured in DMEM (11965, GIBCO) with 10% fetal bovine serum (FBS) (16000, GIBCO) and 1% penicillin/streptomycin (P/S) (15070, GIBCO: Penicillin: 10,000 Unit/mL, streptomycin: 10,000 µg/mL) in a 5% CO_2_ humidified atmosphere at 37 °C. The cultured cells were replenished with fresh media twice a week until they reached 80~90% confluence. SOX9 short hairpin RNAs (Sox9 shRNA) (SHCLNG-NM_000346) and Mission lentiviral packaging mix (SHP001) were purchased from Merk. RIPK1 small interfering RNAs (RIPK1 siRNA) (SR305755) were purchased from ORIGENE (Rockville, MD). SOX9 shRNA and RIPK1 siRNA sequences are listed in Supplementary Materials (Table S1). Cells numbering 2774 were lentivirally transduced using pLKO.1-Sox9 plasmid or pLKO.1-control and maintained in DMEM media supplemented with 10% FBS and with additional 1µg/mL puromycin for selection.

### 2.2. Immunohistochemistry and Quantification

The tissues were fixed in 10% formalin, embedded in paraffin wax, and then sectioned into 4 µm using a Shandon cryostat (Thermo Fisher Scientific). The paraffin microsections were deparaffinized in xylene and rehydrated in graded alcohol (Sigma-Aldrich). For immunostaining, the sections were subjected to heat-induced epitope retrieval with citrate buffer (pH 6.0) for 3 min at 121 °C, and then endogenous peroxidase activity was blocked with 0.3% hydrogen peroxide for 10 min at room temperature, followed by washing in a PBS buffer. The sections were treated with serum-free blocking solution (S202386-2, Agilent, Santa Clara, CA, USA) for 20 min at room temperature to block non-specific binding and then incubated with the indicated antibody against SOX9 (Santa Cruz Biotechnology), ALDH1 (Santa Cruz Biotechnology) or EpCAM (Santa Cruz Biotechnology) overnight at 4 °C. After washing, the sections were incubated for 30 min at room temperature with HRP-labeled polymer-conjugated secondary antibodies against rabbit IgG or mouse IgG and then counterstained with Mayer’s hematoxylin (Sigma-Aldrich) for 30 sec before dehydration and mounted with Permount (Thermo Fisher Scientific). Virtual slides were created by scanning all stained slides with a ScanScopeAT (Leica Biosystems, Vista, CA, USA) digital slide scanner. All histochemical quantitative analysis was performed by randomly selecting 10 fields of each tissue image. DAB staining intensity corresponding to target protein expression was calculated using the positive pixel counting algorithm of ImageScope software v12 (Leica Biosystems) on the selected images. All human tissue samples used in this study were used after approval by the Institutional Review Board (IRB) of the Samsung Medical Center (IRB approval code: 2007-07-056).

### 2.3. Immunofluorescence (IF)

For immunofluorescence, the cells were seeded in a 60-millimeter dish (SPL Life Sciences, Seoul, Korea), fixed in 4% paraformaldehyde for 10 min, permeabilized in 1% Triton X-100 for 10 min, and blocked with 3% BSA in PBS for 30 min at room temperature. The fixed cells were incubated with indicated primary antibody (Santa Cruz). Secondary Alexa Fluor 488-conjugated or Alexa Fluor 568-conjugated antibodies (Thermo Fisher Scientific) were used for visualization, and nuclei were counterstained with DAPI (Vector Laboratories, Burlingame, CA, USA). Confocal fluorescence images were produced on an LSM 700 scanning microscope (Zeiss, Oberkochen, Germany) using ZEN 2.3 lite software (Zeiss).

### 2.4. Quantitative Real-Time PCR

RNA from 2774-vec and 2774-sh-SOX9 cell lines was extracted using an RNeasy mini kit (74104, Qiagen, Hilden, Germany) with RNase-Free DNase set (79254, Qiagen), and mRNA was reverse transcribed using the RT² first strand kit (330404, Qiagen). qRT-PCR was conducted using a standard protocol for RT² SYBR Green qPCR Master Mix (330503, Qiagen) with each primer set on an ABI 7900HT Fast Real-Time PCR System (Applied Biosystems, Waltham, MA, USA). Genes for normalization of qRT-PCR were determined by screening Sox9 stem cell marker-related genes and housekeeping gene GAPDH. Primer sequences for qRT-PCR synthesized and purified from Bioneer (Daejeon, Korea) are listed in Supplementary Materials Table S2.

### 2.5. Immunoblotting and Co-Immunoprecipitation

The cells were lysed in an ice-cold RIPA buffer (1% NP-40, 1% sodium deoxycholate, 0.15 M sodium chloride, 0.01 M sodium phosphate (pH 7.4), 2 mM EDTA, 50 mM NaF, 0.2 mM sodium orthovanadate, 40 mM HEPES (pH 7.4), 0.7% CHAPS, 1% sodium dodecyl sulfate (SDS) containing 1× protease inhibitor cocktail (Roche, Penzberg, Germany), and 1× phosphatase inhibitors (Roche)) for 10 min at 4 °C. The homogenized samples were centrifuged at 13,000 rpm for 10 min at 4 °C, and 25 µg of protein was resolved by SDS-PAGE and transferred onto the PVDF membrane. The membrane was blocked in 5% skim milk in TBST, incubated with appropriate primary antibodies, probed with secondary antibodies, and then detected in ECL substrate solutions (Bio-rad, Hercules, CA, USA) according to the manufacturer’s protocol. For co-immunoprecipitation, entire cell lysates were incubated with anti-SOX9 or anti-RIPK1 antibody overnight at 4 °C. Immunocomplexes were immunoprecipitated using protein A/G agarose beads (Santa Cruz Biotechnology, Dallas, TX, USA) for 2 h at 4 °C with gentle stirring. The beads were washed three times with lysis buffer and boiled in 50 μL of 1× SDS sample buffer for 5 min at 95 °C. After centrifugation, precipitated proteins were separated by SDS-PAGE and electrophoretically transferred onto a PVDF membrane. Then, co-immunoprecipitated RIPK1 or SOX9 was detected by immunoblot analysis with its respective antibody and detected as described above [18].

### 2.6. Cell Cycle Assay

In order to confirm the degree of cell cycle arrest in 2.5 µM cisplatin-treated cells, 2774 and 2774-sh-SOX9 cells were plated in 6-well plates and incubated in DMEM with or without cisplatin. After 48 h incubation, the cells were washed with ice-cold 1× PBS and resuspended in 0.6 mL 1× PBS. Then, 1.4 mL cold ethanol was added into the cells, followed by fixation at −20 °C for 4 hr. Subsequently, the cells were centrifuged at 2000 rpm for 5 min and washed once with PBS containing 1% FBS. The cells were re-suspended in 1 mL PBS containing 0.1 mg/mL RNase, 50 µM/mL PI, and 0.05% Triton X-100. The mixture was gently mixed, kept in a dark place, and incubated at 37 °C for 30 min. Finally, the cells were re-suspended in 500 µL 1× PBS and prepared for the FACS assay. The cell cycle profiles were analyzed using a BD FACSVerse (BD Biosciences) with FACSuit software. At least 10,000 cells in each sample were analyzed to obtain a measurable signal. All measurements were performed using the same instrument settings.

### 2.7. Apoptosis

Both 2774-control and 2774-shSOX9 cells were seeded onto a 6-well plate and cultured at 37 °C overnight to reach approximately 50% confluence. Some wells were treated with cisplatin to a final concentration of 2.5 µM cisplatin. The cells were collected 48 h after cisplatin treatment and analyzed using a BD FACSVerse with Annexin-V staining kit according to the manufacturers’ instructions.

### 2.8. Viability Assay/Proliferation Assay

The effect of cisplatin on the viability of both 2774-control and 2774sh-SOX9 cells was determined using the EZ-Cytox Cell Viability Assay kit (Daeil Lab service, Seoul, Korea) in accordance with the manufacturer’s instructions. Each cell line was cultured in a 96-well microplate at 2.5 × 10^3^ cells per well in triplicate in a final volume of 100 µL DMEM containing 2.5 µM cisplatin under the conditions described. DMSO was used as the vehicle control. After 0 h, 24 h, 48 h, and 72 h incubation, 10 µL of EZ-Cytox was added, followed by incubation for 60 min at 37 °C and measurement of the absorbance at 450 nm using an ELISA reader, SpectraMax ABS Plus (Molecular Devices, San Jose, CA, USA). For the determination of cell viability in RIPK1siRNA or control siRNA-transfected 2774 cells, cells were seeded at 10,000 cells/well in a 96-well plate, allowed to adhere overnight, and treated with cisplatin for 72 h. Cell proliferation inhibition was determined via Cell Titer Glo (Promega, Madison, WI, USA), according to the manufacturer’s protocol. The detected luminescent signal was used to calculate the percentage of surviving cells for comparison with that of the control.

### 2.9. Xenograft Experiments

Five-week-old female Balb/C mice (Orient Bio, Seongnam, Korea) were obtained and acclimated for one week in the animal facility before the experiments were conducted. Each count of 1 million cells of 2774 and 2774-sh-SOX9 was resuspended in 100 µL Matrigel/HBSS (1:1 mixture, BD) and injected subcutaneously into the right flank of mice as a xenograft tumor model. After implantation, the animals (*n* = 4 per group) were monitored daily, tumor weight was measured, and tumor volume was measured twice a week using a digital caliper and calculated according to the following formula: volume = (length × width × width) × 0.5. Mice were sacrificed after 20% weight loss or if showing signs of significant distress or loss of limb or motor function. The tumor tissues obtained from the mouse flank were fixed in 10% buffered formalin solution for histological analysis. All procedures were approved (Approval code: 20150520001) by the Samsung Medical Center Institutional Animal Care and Use committee and conducted in accordance with AAALAC International Guidelines and the United States Animal Welfare Act.

### 2.10. Sphere Formation Assay

Cells of type 2774-vec and 2774-sh-SOX9 were cultured in defined serum-free medium composed of DMEM: F12 medium (1:1 mixture, 11320, GIBCO) with the addition of 10 ng/mL of epidermal growth factor (EGF) (236-EG, R&D Systems, Minneapolis, MN, USA), 10 ng/mL of basic fibroblast growth factor (bFGF) (233-FB, R&D Systems), and B27 supplement (17504, GIBCO). The cultured cells were plated in an Ultra-Low Attachment 96-well plate (3471, Corning, Somerville, MA, USA). After 14 days of incubation, spheroids were captured using a microscope (TE2000U, Olympus, Tokyo, Japan), and spheroid number and area were determined by the ZEN program (Zeiss).

### 2.11. Invasion Assay

Cell invasiveness was measured using Matrigel-precoated Transwell plates (8-μm pore size; 3422, Corning Costar, NY, USA). Control and shSOX9 cells were seeded at 1 × 10^5^ cells onto these plates, whereas MDAH 2774 cells were transfected with human RIPK1 siRNA of control siRNA for 48 h, trypsinized, counted, and added to the upper chambers of the Transwell plates. Medium containing 10% FBS was added to the lower chambers and acted as the chemoattractant. After incubation for 24 h, the filter was gently removed from the chamber, and noninvasive cells on the upper surface were removed by wiping with a cotton swab. The cells that invaded the Matrigel and attached to the lower surface of the filter were fixed in 100% methanol and stained with 0.1% crystal violet. These were captured at ×200 magnification under a light microscope (BX51, Olympus) and invaded cells were counted using the ZEN program (Zeiss). Data were expressed as the mean ± SD from three independent experiments.

### 2.12. ALDEFLUOR Assay

The ALDH activity of cells was measured using the ALDEFLUOR™ Kit (STEMCELL, Vancouver, BC, Canada) according to the protocol provided by the manufacturer. A total of 1 × 10^6^ cells were resuspended in ALDEFLUOR assay buffer, 5 µL of DEAB inhibitor was added to the control tube, and 5 µL of activated ALDEFLUOR reagent was added to the test sample tubes. After mixing, 500 µL of the suspension was removed and placed in the control tube with the inhibitor. After incubation for 50 min at 37 °C, cell pellets were resuspended in the 500 µL ALDEFLUOR buffer, and samples were analyzed on the BD FACSVerse.

### 2.13. Yeast Two-Hybrid (Y2H) Screening and Quantitation of Interaction

For bait construction with the human SOX9 gene, a cDNA fragment encoding full-length human SOX9 was obtained by using PCR with indicated specific primers and inserted into the pGilda/LexA yeast shuttle vector with EcoRI and XhoI sites. Three truncated fragments (Met^1^-Phe^204^, Lys^205^-Ser^386^, and Ser^387^-Pro^509^) of SOX9 were inserted into the pGilda/LexA vector utilizing EcoRI and XhoI enzyme sites, respectively. The bait pGilda/LexA-SOX9 (full) and three truncated mutants were transformed according to modified lithium acetate protocols [19]. The human receptor-interacting serine/threonine-protein kinase 1 (hRIPK1) was inserted with cDNA fragments encoding a full-length gene into the pJG4-5 activation vector utilizing EcoRI and XhoI enzyme sites in B42 fusion proteins (Clontech, Palo Alto, CA, USA). Three truncated motifs (Met^1^-Asp^300^, Val^301^-Thr^579^, and Thr^580^-Asn^671^) were inserted into the pJG4-5 activation vector to generate B42 fusion proteins with EcoRI and XhoI enzyme sites. The primer sequences used to clone all constructs are listed in Supplementary Materials Table S3. hRIPK1 (full) and three truncated plasmid constructs encoding pJG4-5 fusion proteins were transformed into yeast competent cells that contained pGilda/LexA-Sox9 (full) and three truncated mutants, with all transformants selected based on tryptophan prototrophy (plasmid marker) in synthetic medium (Ura^−^, His^−^, and Trp^−^) including 2% (*w*/*v*) glucose. Entire transformants were pooled and respread on the synthetic medium (Ura^−^, His^−^, Trp^−^, and Leu^−^) involving 2% (*w*/*v*) galactose to stimulate the transformed cDNA. Yeast cells growing on the selection media (SD) were retested on the synthetic medium (Ura^−^, His^−^, Trp^−^, and Leu^−^) including 2% galactose (inducing condition) and 2% glucose (non-inducing condition) to confirm dependency of their growth on the presence of galactose. Then, selected transformants were validated according to the previously described protocol [19]. The binding activity of the interaction was estimated by an ONPG β-galactosidase analysis according to the protocol reported [19].

### 2.14. Statistical Analysis

The Wilcoxon rank sum test was used to evaluate the significance between target proteins and metastasis, and Spearman’s correlation test was used to analyze the correlation between the target proteins in the metastatic group. All other results were statistically analyzed using Student’s T-test. The following *p* values were considered to be statistically significant: *p* value ≤ 0.05 (*), *p* value ≤ 0.01 (**), and *p* value ≤ 0.001 (***). 

## 3. Results

### 3.1. SOX9 Protein Expression Is Correlated with Lymph Node Metastasis and ALDH1 Protein Expression in HGOC

**Figure 1:**
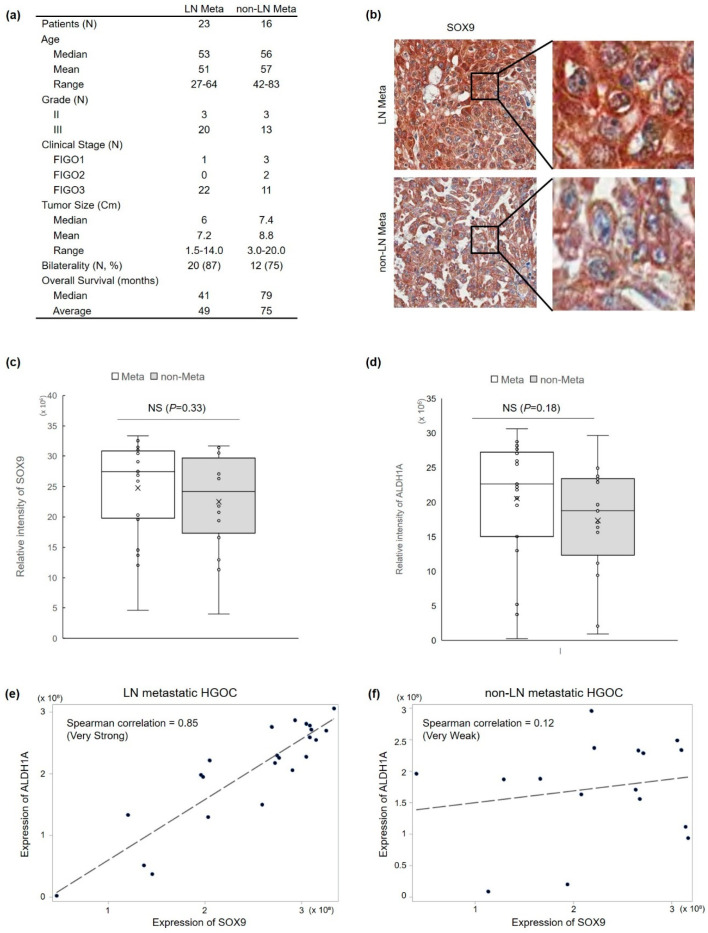
Overexpression of cytoplasmic-SOX9 and ALDH1 proteins in HGOC was associated with lymph node (LN) metastasis. (**a**) Clinical-pathological characteristics of ovary cancer specimens used in this study. (**b**) Representative images of immunohistochemical staining of SOX9 and ALDH1 proteins in HGOC. (**c**,**d**) Wilcoxon ranking sum analysis showed no significant (NS) association between LN metastasis and the expression level of SOX9 protein (**c**) or ALDH1 protein (**d**). (**e**,**f**) Spearman’s correlation analysis confirmed a very strong correlation between SOX9 and ALDH1 expression levels in LN metastatic HGOC group (**e**) and no correlation between the two protein levels in non-metastatic HGOC group (**f**).

Accumulating experimental and clinical data have suggested an association between cancer stemness and ovarian metastasis [4], and overexpression of transcription factor SOX9 protein was suggested in cancer malignancies, recurrence, and drug resistance in different types of cancers including ovarian cancer [15,16]. In order to examine whether the expression of SOX9 and aldehyde dehydrogenase 1 (ALDH1) proteins is associated with lymph node metastasis in HGOC, we used immunohistochemistry to analyze SOX9 and ALDH1 protein expression in 39 HGOC samples with or without lymph node metastasis (Figure 1a). Interestingly, unlike the nuclear localization of transcription factor SOX9 protein in other types of cancer tissues, we observed the preferential cytoplasmic localization of SOX9 protein in HGOC (Figure 1b). Quantitative analysis of immunohistochemical staining showed no significant correlation between lymph node metastasis and the expression of SOX9 or ALDH1 stemness marker protein, respectively (Figure 1c,d). However, a strong correlation between the level of SOX9 protein expression and the level of the cytoplasmic cancer stem cell marker ALDH1 expression was found only in the lymph node metastatic HGOC group (Figure 1e). In contrast, there was no correlation between the expression level of the two proteins in non-metastatic HGOC (Figure 1f). Next, in order to address the specificity of these results, the expression of EpCAM protein, another membrane-localized stem cell marker, was examined by immunohistochemistry with serial sections of the same slides obtained from the HGOC group with lymph node metastasis. There was no significant correlation between SOX9 protein and EpCAM protein expression in the lymph node metastatic HGOC group (Figure S1). Immunohistochemistry results with clinical data suggested the specificity of a strong correlation between SOX9 and ALDH1 in metastatic HGOC.

### 3.2. SOX9 Silencing Abrogates Platinum Resistance and Tumorigenesis in HGOC Cells

**Figure 2:**
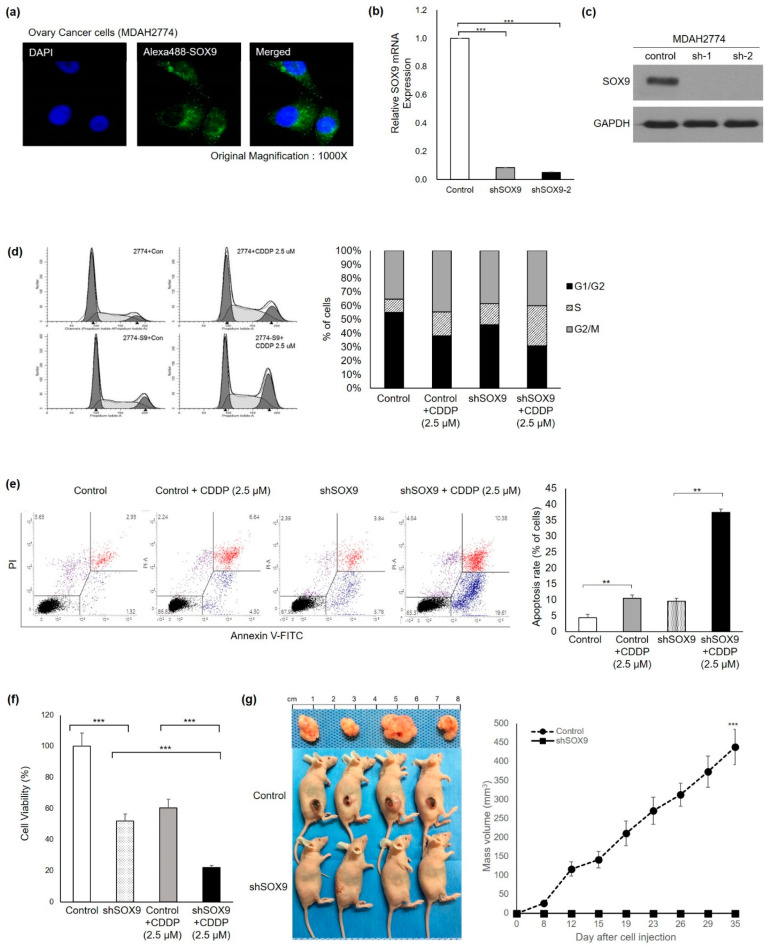
SOX9 silencing induces cancer cell death in vitro and in vivo. Knockdown of SOX9 in MDAH-2774 ovarian cancer cells increase anticancer drug responsiveness and inhibits tumorigenesis. (**a**) High expression of cytoplasmic SOX9 in MDAH-2774 was confirmed by immunocytochemistry. Alexa-488 (green) was used as a secondary antibody to visualize SOX9 protein expression. DAPI was used to stain the nucleus. (b and c) Stable knockdown of SOX9 mRNA and protein expression by lentivirus containing vector alone and SOX9-specific shRNAs (sh1 and sh2) was confirmed by qRT-PCR (**b**) and immunoblot analysis (**c**). (**d**) SOX9 silencing markedly altered the cell cycle, resulting in increased S phase and decreased G1 phase. (**e**) SOX9 silencing markedly induced cancer cell death in CDDP-resistant 2774 cells. (**f**) Inhibition of ovarian cancer cell proliferation by SOX9 silencing. Ovarian 2774 cell proliferation was analyzed by EZ-Cytox assay after a 72 h CDDP treatment. A significant reduction in cell viability by CDDP treatment in SOX9 knockdown cells. (**g**) Complete suppression of tumor formation by SOX9 knockdown. Stable 2774 cells harboring vector and SOX9-specific shRNA were injected into Balb/c mice, and tumor growth was analyzed for 35 days. Knockdown of SOX9 failed to form a detectable tumor mass. All data are presented as the mean ± SD of three independent experiments. ** *p* < 0.01, and *** *p* < 0.001 indicate a significant difference.

In order to explore the unusual cytoplasmic localization of SOX9 protein in lymph node metastatic HGOC, we screened human ovarian cancer cells for SOX9 expression level and its subcellular localization. We selected 2774 cells due to its high SOX9 mRNA and cytoplasmic SOX9 protein expression found in HGOC samples and human ovarian cancer cell lines. Consistent with high cytoplasmic SOX9 protein expression, a predominant cytoplasmic localization of SOX9 protein was detected in MDAH 2774 cells by confocal microscopy after fluorescent immunocytochemistry (Figure 2a and Figure S2). In order to explore the impact of SOX9 gene knockdown in 2774 cells with endogenous high cytoplasmic expression of SOX9, a lentiviral vector containing non-specific shRNA or lentiviral vector expressing SOX9shRNA was infected into parent 2774 cells. Greater than 90% suppression of SOX9 mRNA and protein expression was confirmed by RT-PCR and immunoblot analyses (Figure 2b,c). First, we examined the effect of SOX9 gene silencing on the cell cycle by FACS analysis. Representative plots of FACS analysis demonstrated that SOX9 gene silencing enhanced the S phase of the cell cycle by 1.9-fold in SOX9shRNA-silenced 2774 cells (2774-shSOX9) compared to non-specific shRNA control vector 2774 cells (2774-control) after CDDP treatment (Figure 2d). Second, in order to assess the effect of SOX9 gene silencing on anticancer CDDP sensitivity, both cell proliferation assay and cell apoptosis assay were performed with 2774-control and 2774-shSOX9. In order to determine the CDDP concentration for apoptosis analysis, we chose a 2.5-millimolar concentration based on a previous experiment (Figure S3). Apoptosis analysis revealed that the 2.5 mM CDDP treatment resulted in 10.4% apoptosis in 2774 control cells, which reflected the drug resistance expected in 2774 cells. In contrast, SOX9 gene silencing significantly enhanced apoptosis in 2774-shSOX9 cells upon CDDP treatment, indicating enhancement of drug sensitivity by up to 37.5% (3.6-fold, Figure 2e). Next, we determined the effect of SOX9 silencing in cancer cell proliferation. Ovarian cancer cell proliferation was analyzed by using the EZ-Cytox Cell Viability Assay after 72 h CDDP treatment. Non-specific shRNA control 2774 cells showed that expected cell growth and CDDP treatment of 2774-control cells moderately reduced cell survival. In contrast, SOX9 gene knockdown in 2774-shSOX9 cells significantly suppressed cell viability upon CDDP treatment (Figure 2f). These results suggest that knockdown of the SOX9 gene not only inhibits cell survival but also induces marked cell apoptosis after anticancer drug CDDP treatment in 2774-shSOX9 cells. Third, in order to evaluate the effect of SOX9 gene silencing in ovarian tumorigenesis in vivo, we conducted an in vivo xenograft mouse experiment. A sizable tumor mass was observed only in the control group of mice injected with 2774-control, whereas experimental mice group injected with 2774-shSOX9 showed no tumor mass formation over 35 days after subcutaneous injection (Figure 2g), suggesting complete eradication of tumor in vivo. These in vitro and in vivo results demonstrated that SOX9 expression was required for the prevention of cancer cell death as well as for the promotion of cancer cell proliferation, consequently resulting in tumor growth. Thus, SOX9 gene silencing in these experiments revealed not only complete suppression of cancer cell proliferation but also effective induction of cancer cell death, resulting in complete suppression of tumor growth in an in vivo xenograft model.

### 3.3. Silencing of Sox9 Abrogated the Cancer Stemness Properties in Ovarian Cancer Cells

**Figure 3:**
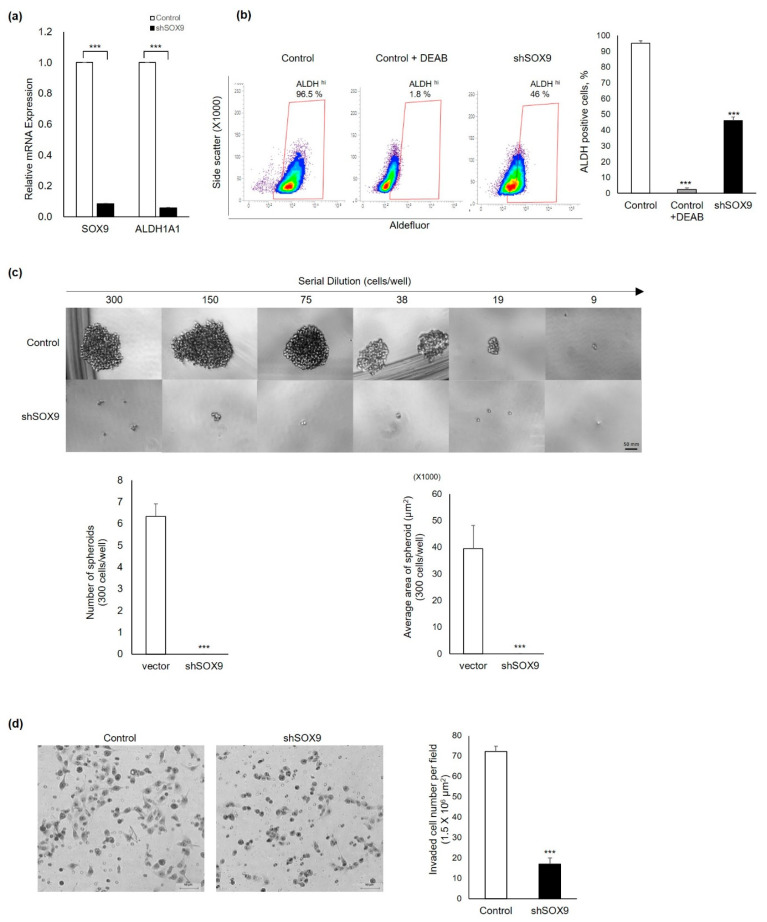
Silencing of SOX9 in human ovarian 2774 cancer cells with high SOX9 expression resulted in loss of cancer stem-like properties. (**a**) SOX9 silencing strongly suppressed ALDH1A1 mRNA expression measured by qRT-PCR analysis. Control 2774 cells and 2774-Sox9sh cells harboring vector alone and SOX9- specific shRNA(sh1) were harvested to extract the total RNA for qRT-PCR assay to measure ALDH1A1 mRNA expression. (**b**) Reduction in ALDH activity by SOX9 knockdown. ALDH1 activity was analyzed by Aldefluor assay. (**c**) Spheroid formation was blocked by SOX9 knockdown in 2774 cells. Using 3D cell culture conditions, 2774-control cells formed discrete and compact spheres, whereas 2774-Sox9shRNA cells failed to form spheres. (**d**) Suppression of cancer cell invasion by SOX9 knockdown. Control vector 2774 and 2774-shSOX9 cells were seeded onto Transwells on Matrigel-coated Transwells. The number of invaded cells was counted under a light microscope, and mean values were plotted. Data present the mean ± SD of three independent experiments. *** *p* < 0.001 indicate significant difference.

Since cancer stemness was associated with ovarian cancer progression and prognosis, next, we explored the effect of SOX9 gene silencing on the cancer stemness of ovarian cancer cells. We analyzed mRNA expression and activity of a well-characterized cancer stemness marker, aldehyde dehydrogenase1alpha1(ALDH1A1), which was reported to be associated with platinum resistance and poor prognosis [8]. Silencing the SOX9 gene reduced both ALDH1A1 mRNA expression and its activity (Figure 3a,b). These results revealed inhibition of cancer stemness by SOX9 silencing. Next, to confirm the marked reduction in ALDH1A1 activity by SOX9 gene silencing, a sphere formation assay was utilized to verify functionally the effect of SOX9 silencing in CSCs with high potential self-renewal. The non-specific shRNA control vector and S9shRNA-infected cultured 2774 cells were serially diluted and seeded at the indicated cell numbers for spheroidal cell formation. Spheroid-shaped cell clusters were formed in all wells cultured from 19 to 300 cell numbers of non-specific shRNA control vector-infected 2774 cells, whereas spheroid cell mass was not formed in wells cultured with any number of S9shRNA-infected 2774 cells (Figure 3c). Since lymph node metastasis is associated with cancer cell migration, we explored whether the SOX9 gene regulated the invasiveness of cancer cells by using a Transwell invasion assay. As anticipated, silencing the SOX9 gene significantly reduced the invasion of 2774 cells (Figure 3d). Quantitative analysis showed that silencing of the SOX9 gene inhibited cancer cell invasion by 76% compared to control non-specific shRNA vector-infected 2774 cells. Taken together, our findings demonstrated that SOX9 silencing markedly reduced the expression and activity of cancer stem cell marker ALDH1A1 and inhibited cancer cell migration.

### 3.4. Identification of Serine/Threonine-Protein Kinase 1 (RIPK1) as a Novel SOX9-Interaction Protein

**Figure 4:**
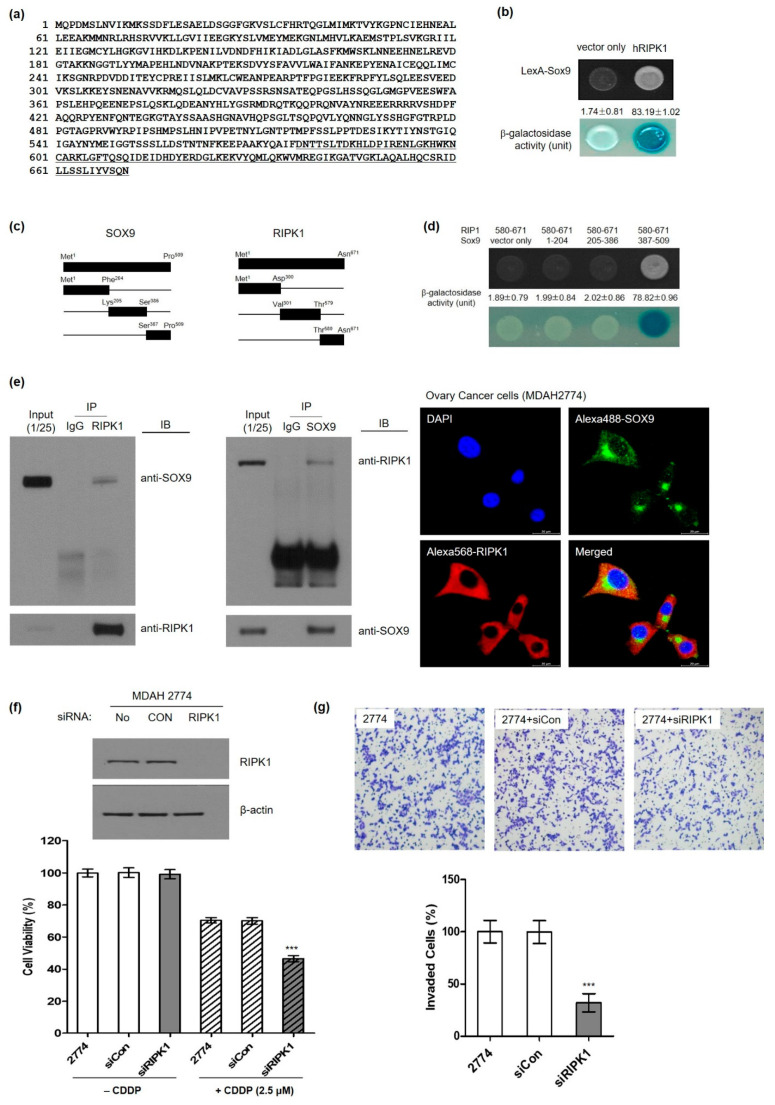
Stem cell factor SOX9 interacts with a cell death regulator RIPK1. (**a**) The amino acid sequence of human RIPK1 is indicated by single-letter abbreviations. The underlined amino acid sequence is the translated hRIPK1 protein identified from the yeast two-hybrid assay. (**b**) Identification of RIPK1 as a novel SOX9 by yeast 2-hybrid (Y2H) screening. Yeast transformants were assayed for their ability to grow on medium lacking leucine at 30 °C and for galactosidase expression through the formation of a blue colony on the plate containing X-gal. (**c**) A schematic representation of the cDNA constructs for both SOX9 and RIPK1. (**d**) Identification of the interacting domains between SOX9 and RIPK1 by a Y2H analysis. Analysis with the indicated deletion constructs of SOX9 and RIPK1 by the yeast two-hybrid analysis. The C-terminal of SOX9 protein (Ser^387^-Pro^509^) interacted with the domain comprising the amino acid region of RIPK1 protein (Thr^580^-Asn^671^) by Y2H assay. (**e**) (**Left**) Endogenous SOX9 and RIPK1 interaction in human cells. HEK 293 cell lysates were prepared in lysate buffer and then subjected to immunoprecipitation with anti-IgG, anti-RIPK1, or anti-SOX9 antibody, followed by immunoblot analysis with the respective antibody. Expression of RIPK1 and SOX9 was confirmed by immunoblot analysis with the respective antibody. (**Right**) Cytoplasmic co-localization of SOX9 and RIPK1 in human ovarian cancer cells. MDAH 2774 cells were incubated with anti-SOX9 and anti-RIPK1 antibody and then subjected to indirect immunofluorescence staining with Alexa Fluor 568- and 488-conjugated secondary antibody, respectively. The subcellular co-localization of SOX9 and RIPK1 was examined using confocal microscopy. The co-localization of SOX9 and RIPK1 is shown in the merged images. Scale bar, 20 µm. (**f**) Silencing of RIPK1 dramatically increased CDDP sensitivity. MDHA 2774 cells were transiently transfected with control siRNA or RIPK1 siRNA for 24 h and then treated with 2.5 µM CDDP for 72 h. Cell viability was measured by cell titer proliferation assay. (**g**) Suppression of cell migration by RIPK1 silencing. MDAH 2774 cells were transfected with human RIPK1 siRNA or control siRNA and then then processed relative to invasion assay using Matrigel-coated Transwells. The number of invaded cells was counted, and mean values were plotted. Data present the mean ± SD of three independent experiments. *p* < 0.001 (***) indicates a significant difference.

Our clinical pathological analysis showed a strong correlation between SOX9 protein expression and ALDH1 protein expression only in metastatic HGOC samples, indicating an association among SOX9 expression, platinum drug resistance, and lymph node metastasis. We found unexpected preferential cytoplasmic subcellular localization of transcription factor SOX9 in HGOC samples, and silencing SOX9 induced cancer stem cell death. These findings suggest the active role of cytoplasmic SOX9 protein in cancer stem cell death. To investigate a yet unidentified role of preferential cytoplasmic SOX9 protein in HGOC and to explore an underlying molecular mechanism, we aimed to identify a candidate cytoplasmic interacting protein by yeast 2-hybrid (Y2H) assay. We identified a putative protein, and a homology search in GenBank utilizing the BLAST program revealed four of five plasmid cDNA fragments screened by Y2H-encoded human receptor-interacting serine/threonine-protein kinase 1 (RIPK1) (accession number: NM_003804.6) (Figure 4a). A positive interaction between SOX9 and RIPK1 was verified both by cell growth and a β-galactosidase assay. As shown in Figure 4b, β-galactosidase activity was shown for the interaction between SOX9 and RIPK1 (83.19 ± 1.02), but it was not observed with vector only (1.74 ± 0.81) as the negative control for the assay. In order to further confirm the interaction and to map critical binding domains between SOX9 and RIPK1 proteins, plasmid constructs containing three truncated fragments for both proteins, respectively, were generated (Figure 4c). Three truncated RIPK1 and SOX9 plasmids expressing the indicated domains (Met^1^-Phe^204^, Lys^205^-Ser^386^, and Ser^387^-Pro^509^) and (Met^1^-Asp^300^, Val^301^-Thr^579^, and Thr^580^-Asn^671^) were prepared and transformed into EGY48 yeast host strain cells. Using the same Y2H assay, the indicated truncated regions of SOX9 and RIPK1 were transformed into EGY48 yeast competent cells. As shown in Figure 4d, the β-galactosidase assay demonstrated that the critical RIPK1 domain that is critical for binding with SOX9 protein resided within Thr^580^-Asn^671^ (Figure 4d). The RIPK1 serine/threonine kinase protein is a cytoplasmic protein with well-documented cell death associated functions. Interestingly, the C-terminus of RIPK1 (Thr^580^-Asn^671^) containing the death domain (DD) showed potential interaction with SOX9 protein. To support these findings, we performed the Y2H assay using the binding domain of RIPK1 (Thr^580^-Asn^671^) and three truncated SOX9 mutants (Met^1^-Phe^204^, Lys^205^-Ser^386^, Ser^387^-Pro^509^) (Figure 4d). Only yeast cells containing the RIPK1 domain (Thr^580^-Asn^671^) and the SOX9 truncated domain (Ser^387^-Pro^509^) grew on the SD-deficient plate, while yeast cells transformed with other truncated SOX9 regions (Met^1^-Phe^204^ and Lys^205^-Ser^386^) failed to grow. β-galactosidase activity analysis showed an agreement with these results (Figure 4d). Consistent with this result, the C-terminal region of RIPK1 was initially identified from library screening (Figure 4a, underlined). Next, to confirm SOX9-RIPK1 interaction in human ovarian cancer cells, MDAH 2774 cells with high SOX9 and RIPK1 protein levels were subjected to immunoprecipitation followed by immunoblotting. Consistent with the interaction results in a yeast system, a co-immunoprecipitation assay with anti-SOX9 and anti-RIPK1 showed an interaction between them in ovarian cancer cells (Figure 4e, left panel). In addition, we investigated their interaction by confocal imaging analysis after indirect immunofluorescence assay using the same 2774 cells. We observed colocalization of both SOX9 and RIPK1 proteins in the cytoplasm (Figure 4e, right panel), supporting their interaction in human ovarian cancer cells.

In order to investigate the functional significance of SOX9-RIPK1 interaction in ovarian cancer cells, 2774 cells were transfected with RIPK1-specific siRNA or control siRNA, and CDDP sensitivity and cell invasion ability were determined. Deletion of RIPK1 dramatically increased CDDP sensitivity in 2774 cells, as in SOX9 knockdown cells (2774-shSOX9). RIPK1 silencing alone in 2774 cells did not affect cell viability, suggesting a specific role of RIPK1 in CDDP sensitivity (Figure 4f). In addition, 2774 cancer cell migration as determined by Transwell assay was reduced significantly by RIPKI silencing (Figure 4g). These results suggest the critical role RIPK1 plays in SOX9-mediated cancer cell migration and CDDP sensitivity.

## 4. Discussion

The role of SOX9 in the anticancer resistance of CSC and the association of ALDH1 and SOX9 in cancer stem-like properties has been reported by other investigators [20,21]. ALDH1 is a cytoplasmic enzyme that oxidizes aldehydes in cells and has been widely used to characterize stem cells (CSCs) [22]. ALDH1 mediates resistance to chemotherapy via direct involvement of drug metabolism and ROS regulation, and high ALDH activity was reported as a prognostic marker and a therapeutic target in many cancers including ovarian cancer [8,22].

In this study, we found an unusual cytoplasmic expression of SOX9 protein in tumors of metastatic HGOC patients and showed a correlation between the CSC marker ALDH1A1 and SOX9 protein expression in the HGOC with lymph node metastasis (Figure 1). We investigated whether cytoplasmic SOX9 played a role in cell death escape by regulating CSC properties by conducting in vitro and in vivo experiments (Figure 2 and Figure 3). Our experimental results demonstrated that cytoplasmic SOX9 plays a critical role in suppressing cancer stem cell death, as shown by complete suppression of sphere formation in vitro and tumor formation in vivo. The marked induction of cell death to the standard CDDP treatment in human ovarian cancer cells (Figure 2d,e) and complete inhibition of sphere formation (Figure 3c) and cancer cell invasion (Figure 3d) suggest the potential therapeutic effect of SOX9 silencing in human ovarian cancer. However, we did not directly address the therapeutic effect of SOX9 silencing in vivo. Thus, it would be important to evaluate whether SOX9 silencing by SOX9-specific shRNA or anti-sense oligonucleotide (ASO), which can inhibit SOX9 mRNA stability or SOX9 protein translation, is able to reduce tumor growth and metastasis in a xenograft mouse model harboring preformed tumors. Further experiments are required to explore the therapeutic effect of SOX9 silencing and to examine its side effects in ovarian cancer. Consistent with its potential role in cell death, we discovered RIPK1 as a novel binding partner for stem cell factor SOX9 and proposed subcellular localization as a novel SOX9-mediated cell death regulatory mechanism in metastatic HGOC with defective apoptosis machinery.

Consistent with our findings, unusual cytoplasmic SOX9 protein expression has been reported in previous studies. The accumulation of cytoplasmic SOX9 was detected in breast tissues of patients with invasive ductal carcinoma and lymph node metastasis and cytoplasmic SOX9 expression showed a significant correlation with increased cell proliferation of breast tumors [23]. Furthermore, cytoplasmic SOX9 protein showed a direct contribution to poor clinical outcomes associated with breast cancer invasiveness, whereas nuclear SOX9 protein expression was more common in the early stages of differentiation of breast cancer cells [23,24]. These results in breast cancer suggested a specific function of cytoplasmic SOX9 in the regulation of oncogenesis and cancer stem cell properties. A recent publication by Ma et al. reported the crucial role of the SOX9 transcription factor in regulating triple-negative breast cancer (TNBC) growth, survival, and metastasis. The knockdown of SOX9 by siRNA treatment in a TNBC cell line induced TNBC cell death and reduced invasion by regulating gene expression involved in the cell death pathway including RIPK1 by Chip-seq analysis and reduced tumor growth and lung metastasis by SOX9 knockout in an in vivo animal model [25]. They proposed that SOX9 directly binds to the promoter region of death-inducing genes and the promoters of EMT and metastasis-promoting genes in TNBC with high expressions of SOX9 suppressing or upregulating the respective genes. Conversely, in breast cancers with low SOX9 expression, a SOX9-mediated suppressive effect on death-inducing gene expression cannot be exerted, resulting in reduced cell growth, decreased cell survival, EMT, and metastasis.

Our identification of cytoplasmic localization of SOX9 in the HGOC proposes an unidentified role of SOX9 in cell death prevention of cancer stem cells in HGOC. The interaction of stem cell factor SOX9 with RIPK1 in the cytoplasm would contribute to the suppression of cancer cell death and stemness in metastatic ovarian cancer. Based on domain mapping results, interaction of the DD domain of RIPK1 with the transcription activation domain of SOX9 would abrogate not only the transcriptional activation of pro-survival and stemness-related gene transcription by SOX9 transcription factor but also RIPK1-mediated cancer cell death by necroptosis in the cytoplasm, resulting in cancer stem cell survival in HGOC. Considering the abrogation of the apoptotic mechanism in human ovarian cancer cells with stemness, necroptosis might be an alternative pathway for inducing the death of ovarian cancer cells. Prevention of cancer cell death by protein interaction between stemness-regulating SOX9 and RIPK1 contributes an evasion of an apoptotic cell death in devastating ovarian cancers.

Among many regulatory mechanisms involved in the regulation of SOX9 function, protein–protein interaction is important. Our discovery of cytoplasmic RIPK1 as a stem cell factor SOX9-binding partner suggests that cytoplasmic subcellular localization of transcription factor SOX9 in HGOC might be an effective regulatory mechanism of SOX9-mediated cell death escape in HGOC. In addition to a well-established SOX9-mediated transcriptional activation/repression of sets of genes involved in the process of cancer stemness regulation, our SOX9-RIPK1 protein interaction in the cytoplasm is the first such discovery and provides a critical clue for suggesting a mechanism of cancer cell death avoidance in HGOC. In recent years, the pro-death function of RIPK1 was intensively investigated [26,27], and highly selective small molecule inhibitors of RIPK1 kinase activity was shown to be effective in preclinical models and clinical trials [26]. We speculate that interaction of cytoplasmic SOX9 protein with RIPK1 can prevent the availability of death domain of RIPK1 for downstream cell death process involving necrosome formation, thereby resulting in cytoplasmic SOX9-mediated escape of cancer cell death and poor prognosis in metastatic HGOC. Furthermore, domain-mapping results of SOX9-RIPK1 interaction can be applied to design a therapeutic small molecule target for SOX9 protein, which is highly expressed in HGOC. The critical functions of RIPK1 in the regulation of non-apoptotic forms of programmed cell death suggest RIPK1 as alternative therapeutic in cancers with defective apoptosis machinery [26,27,28].

## 5. Conclusions

Our results show a strong association between unusual cytoplasmic SOX9 protein expression and a stem cell marker ALDH1 protein expression only in metastatic HGOC. We demonstrated that the silencing of SOX9 completely suppressed cancer stemness properties in vitro and in vivo. We identified cell death-inducing RIPK1 as an interacting partner of the SOX9 stem cell factor, mapped the death domain of RIPK1 protein as a binding domain of cytoplasmic SOX9, and demonstrated the importance of their protein interaction. Further studies are warranted with follow-up experiments to verify the functional significance of SOX9-RIPK1 interaction and to elucidate the underlying mechanism of SOX9-mediated cell death escape in metastatic HGOC with defective apoptosis machinery.

## Supplementary Materials

The following are available online at https://www.mdpi.com/article/10.3390/cells11030363/s1, Figure S1: Spearman’s correlation analysis showed no correlation between SOX9 and EpCAM expression levels in patients with LN metastatic HGOC. Figure S2: Expression of SOX9 protein in cultured human ovarian and colon cancer cell lines by indirect immunofluorescence staining followed by confocal imaging analysis (left) and immunoblot analysis (right). Figure S3: Determination of CDDP concentration to investigate the effect of SOX9 silencing on CDDP sensitivity by using the EZ-Cytox Cell Viability Assay kit. Figure S4: The non-cropped full-size gel images for immunoblot analysis. Table S1: The shRNA sequences used for construction of SOX9 shRNA lentiviral vector and RIPK1siRNA seqeuences. Table S2: The primer sequences used for quantitative real-time PCR. Table S3: The primer sequences used to clone all fusion constructs for the Y2H assay.
