# Supplementary material for: Stem Cell Factor SOX9 Interacts with a Cell Death Regulator RIPK1 and Results in Escape of Cancer Stem Cell Death
Source: Cells. 2022 Jan 21;11(3):363. doi: 10.3390/cells11030363 (PMC8834197; doi:10.3390/cells11030363)
Supplement: Supplementary file 1 [file cells-11-00363-s001.zip › cells-1521739-supplementary.pdf]

Supplementary data (Oh et al.)

Figure S1: Spearman correlation analysis showed no correlation between SOX9 and Ep CAM expression levels in patients with LN metastatic HGOC,

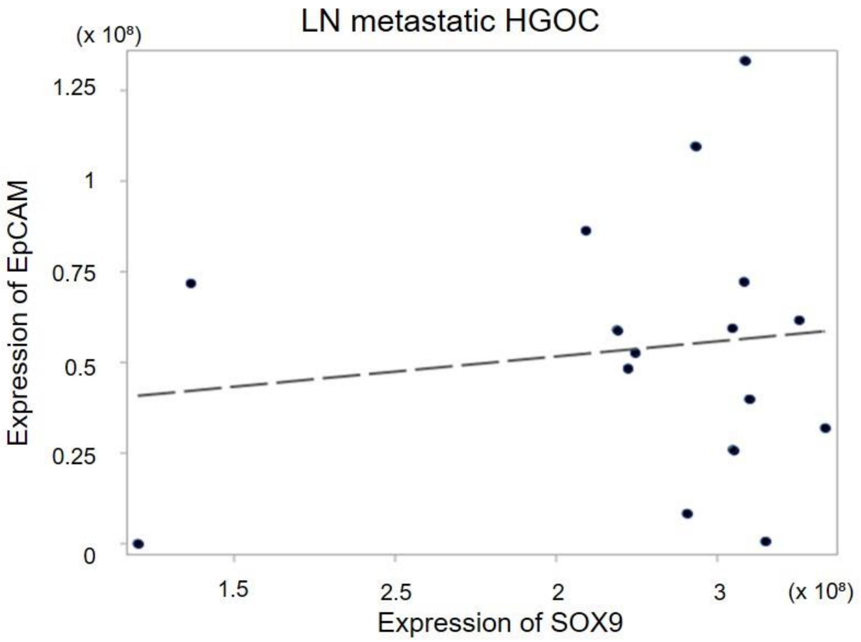

Figure S2: Expression of SOX9 protein in cultured human ovarian and colon cancer cell lines by indirect immunofluorescence staining followed by immunoblot analysis and co nfocal imaging analysis.

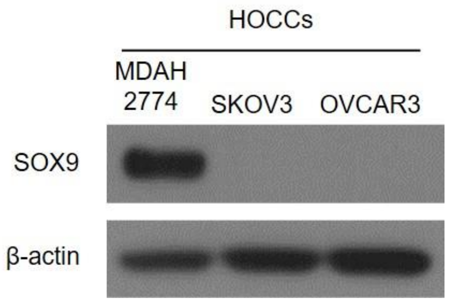

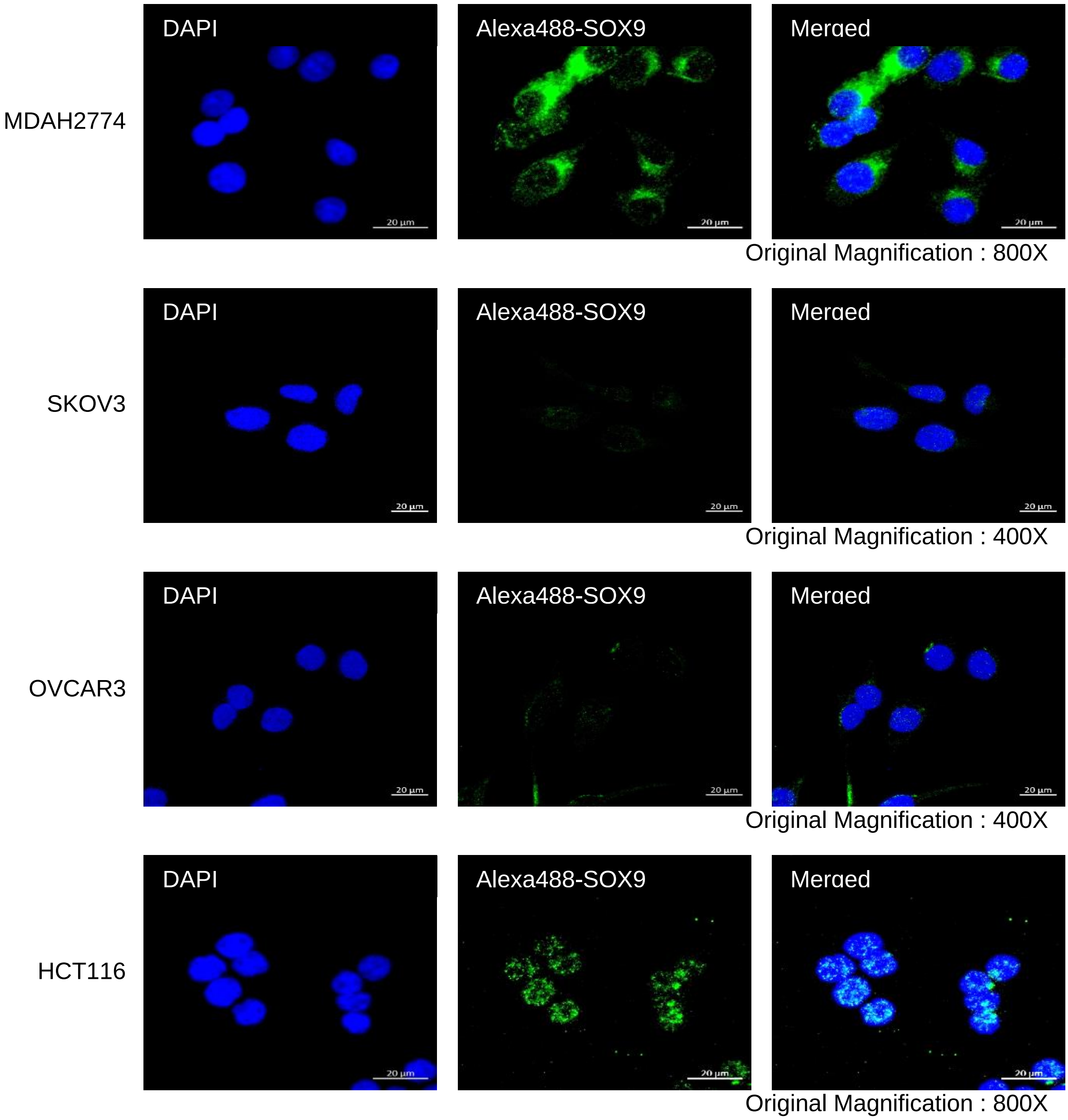
MDAH2774
DAPI
Alexa488-SOX9
Merged
Original Magnification : 800X
SKOV3
DAPI
Alexa488-SOX9
Merged
Original Magnification : 400X
OVCAR3
DAPI
Alexa488-SOX9
Merged
Original Magnification : 400X
HCT116
DAPI
Alexa488-SOX9
Merged
Original Magnification : 800X

Figure S3: Determination of CDDP concentration to investigate the effect of SOX9 silen cing on CDDP sensitivity by using the EZ-Cytox Cell Viability Assay kit.

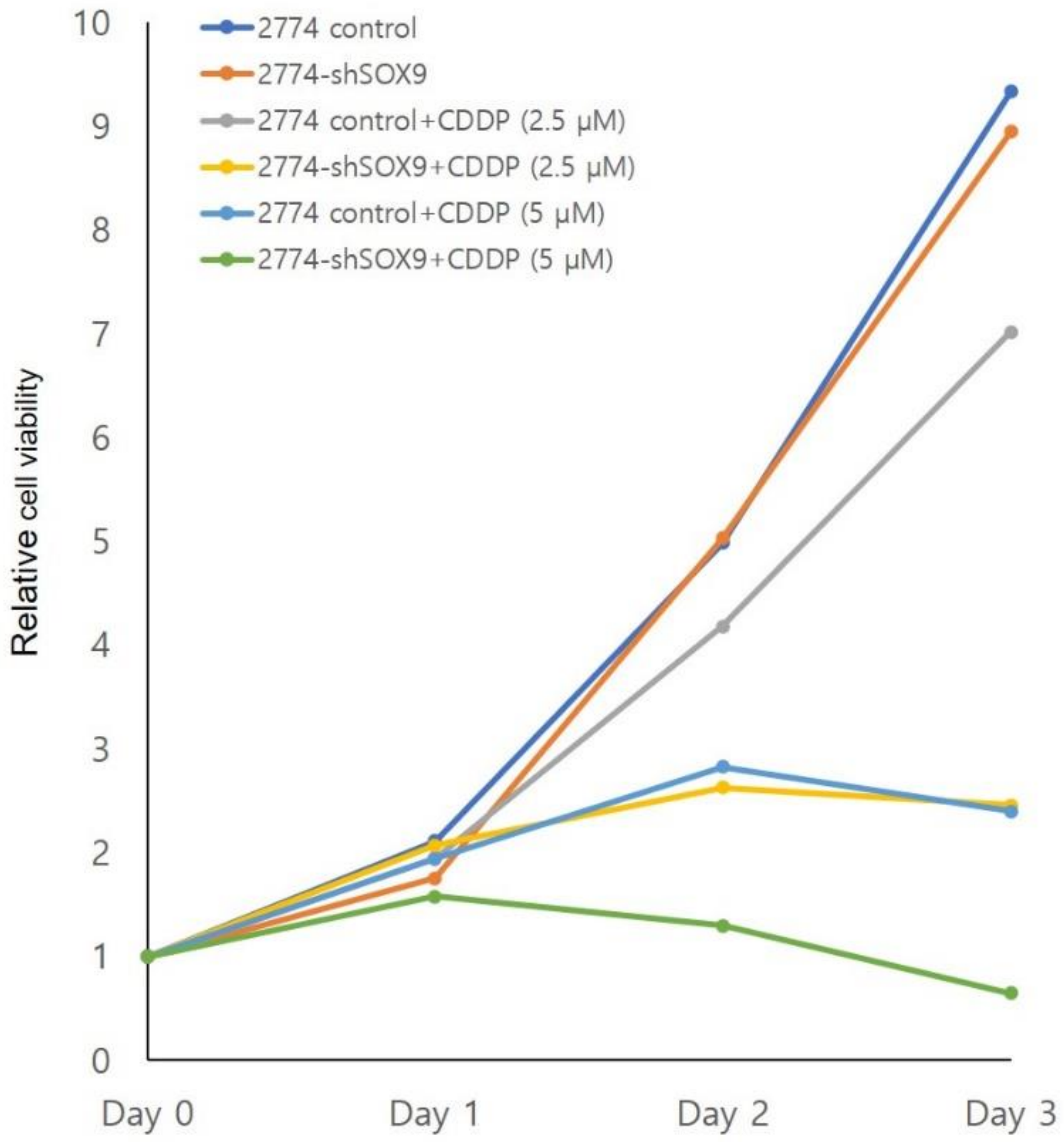

Figure S4: The non-cropped full-size gel images for immunoblot analysis.

Figure 2(c)

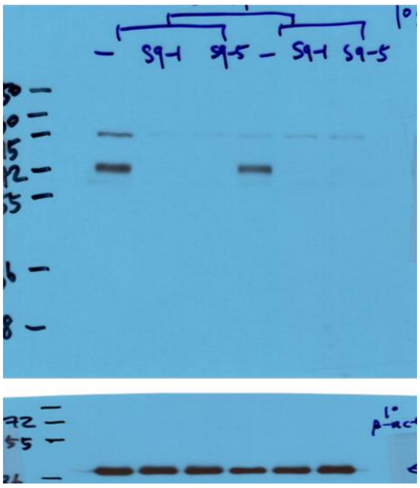

Figure 4(e)

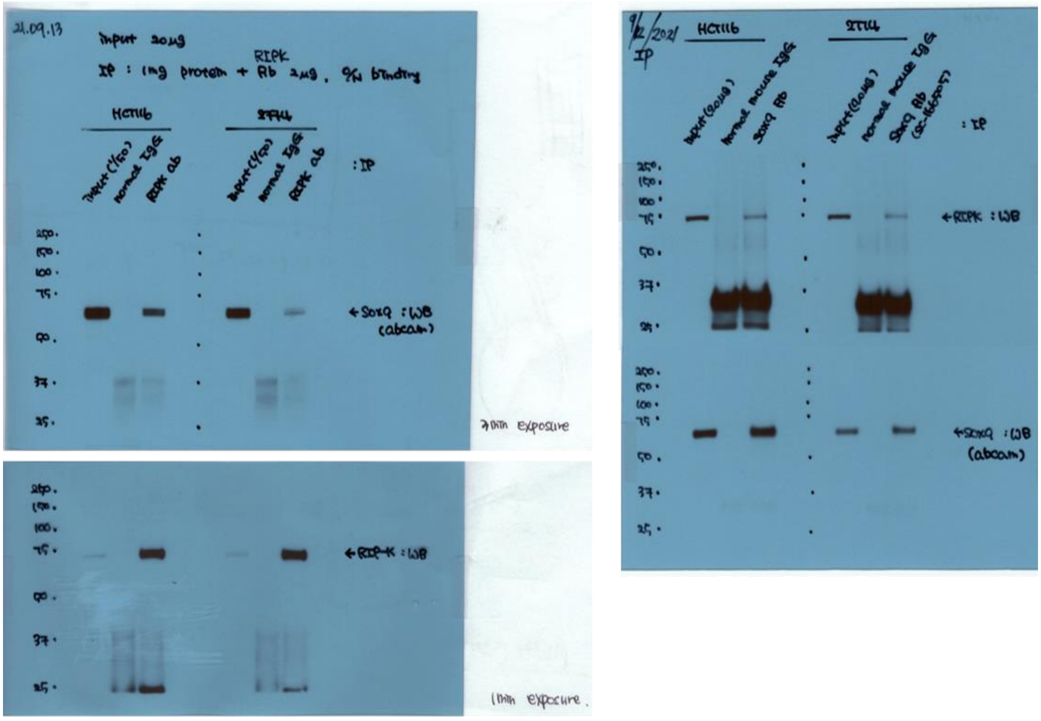

Figure 4(f)

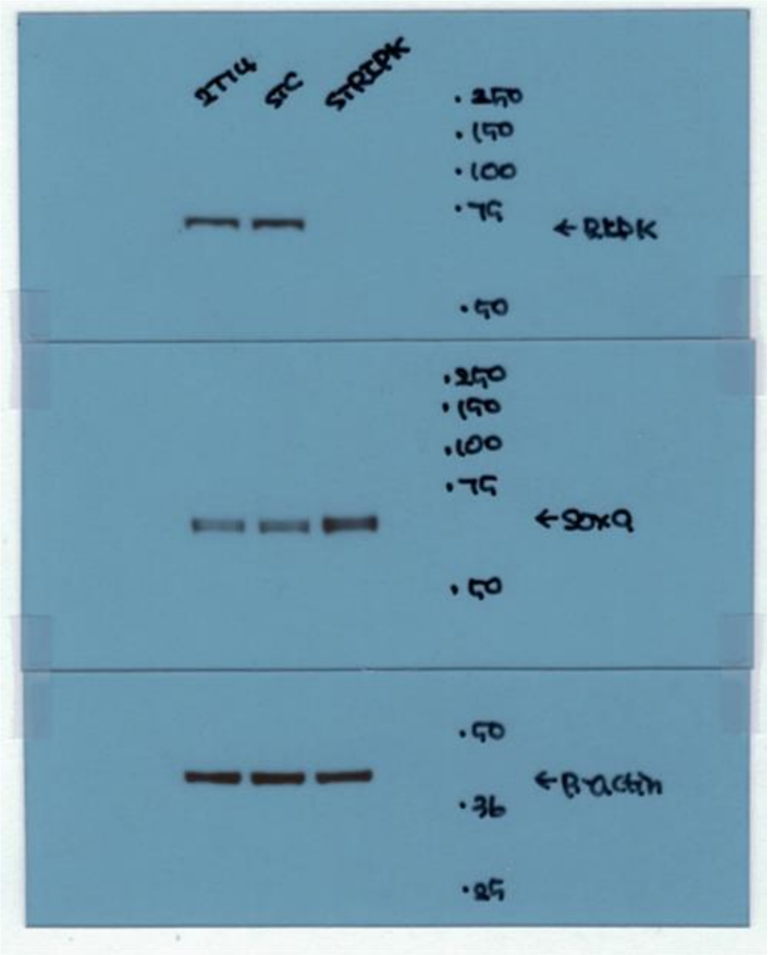

Figure S2

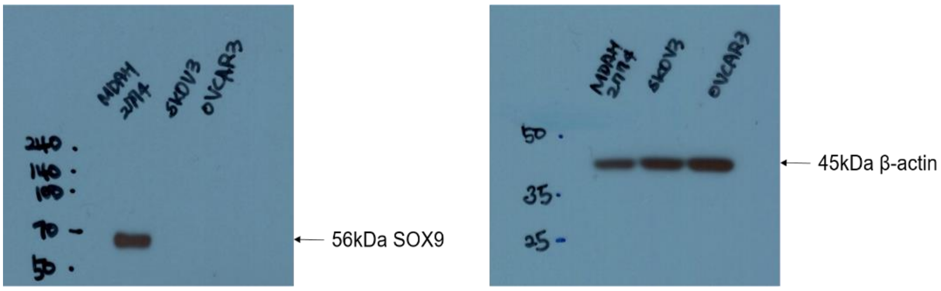

Table S1. The shRNA sequences used for construction of SOX9 shRNA lentiviral vector and RIPK1siRNA sequences for silencing of RIPK1.

Gene Name	TRC Version	Sequence
SOX9	#1	CCGGACTTCTGAACGAGAGCGAGAACTCGAGTTCTCGCTCTCGTTCAGAAGTTTTTTG
	#5	CCGGCTCCACCTTCACCTACATGAACTCGAGTTCATGTAGGTGAAGGTGGAGTTTTTG
**Gene Name**	**Note**	**Sequence**
N/A	siControl	rCrGrUrUrArArUrCrGrCrGrUrArUrArArUrArCrGrCrGrUAT
RIPK1	siRIPK1	rCrCrArCrUrArGrUrCrUrGrArCrGrGrArUrArArArCrArCCT

Table S2. The primer sequences used for quantitative real-time PCR.

Gene Name	Forward Primer sequence	Reverse Primer sequence
GAPDH	5′-GCACCACCAACTGCTTAGC-3′	5′-GGCCATCCACAGTCTTCTG-3′
SOX9	5′-GCGAAATCAACGAGAAACTGG-3′	5′-CAAAGTCCAAACAGGCAGAGA-3′
ALDH1A1	5′-GCACGCCAGACTTACCTGTC-3′	5′-CCTCCTCAGTTGCAGGATTAAAG-3′

Table S3. The primer sequences used to clone all fusion constructs for the Y2H assay.

Gene Construct	Forward Primer sequence	Reverse Primer sequence
Sox9 (1-509)	5′-ATTGAATTCATGAATCTCCTGGACCCC-3′	5′-ATTCTCGAGTCAAGGTCGAGTGAGCTG-3′
Sox9 (1-204)	5′-ATTGAATTCATGAATCTCCTGGACCCC-3′	5′-ATTCTCGAGTCAGAAGATGGCGTTGGG-3′
Sox9 (205-386)	5′-ATTGAATTCAAGGCGCTGCAGGCCGAC-3′	5′-ATTCTCGAGTCAGCTCAGCGTGGTCAG-3′
Sox9 (387-509)	5′-ATTGAATTCAGCGAGCCGGGCCAGTCC-3′	5′-ATTCTCGAGTCAAGGTCGAGTGAGCTG-3′
hRIPK1 (1-671)	5′-CGGGAATTCATGCAACCAGACATGTCC-3′	5′-CGGCTCGAGTTAGTTCTGGCTGACGTA-3′
hRIPK1 (1-300)	5′-CGGGAATTCATGCAACCAGACATGTCC-3′	5′-CGGCTCGAGTTAGTCCTCTTCTACACT-3′
hRIPK1 (301-579)	5′-CGGGAATTCGTGAAGAGTTTAAAGAAA-3′	5′-CGGCTCGAGTTAGGTATTATCAAAGAT-3′
hRIPK1 (580-671)	5′-CGGGAATTCACTAGTCTGACGGATAAA-3′	5′-CGGCTCGAGTTAGTTCTGGCTGACGTA-3′
